# IIS – Integrated Interactome System: A Web-Based Platform for the Annotation, Analysis and Visualization of Protein-Metabolite-Gene-Drug Interactions by Integrating a Variety of Data Sources and Tools

**DOI:** 10.1371/journal.pone.0100385

**Published:** 2014-06-20

**Authors:** Marcelo Falsarella Carazzolle, Lucas Miguel de Carvalho, Hugo Henrique Slepicka, Ramon Oliveira Vidal, Gonçalo Amarante Guimarães Pereira, Jörg Kobarg, Gabriela Vaz Meirelles

**Affiliations:** 1 Laboratório Nacional de Biociências, Centro Nacional de Pesquisa em Energia e Materiais, Campinas, São Paulo, Brazil; 2 Laboratório de Genômica e Expressão, Departamento de Genética e Evolução, Instituto de Biologia, Unicamp, Campinas, São Paulo, Brazil; 3 Laboratório Nacional de Luz Síncrotron, Centro Nacional de Pesquisa em Energia e Materiais, Campinas, São Paulo, Brazil; Swiss Institute of Bioinformatics, Switzerland

## Abstract

**Background:**

High-throughput screening of physical, genetic and chemical-genetic interactions brings important perspectives in the Systems Biology field, as the analysis of these interactions provides new insights into protein/gene function, cellular metabolic variations and the validation of therapeutic targets and drug design. However, such analysis depends on a pipeline connecting different tools that can automatically integrate data from diverse sources and result in a more comprehensive dataset that can be properly interpreted.

**Results:**

We describe here the Integrated Interactome System (IIS), an integrative platform with a web-based interface for the annotation, analysis and visualization of the interaction profiles of proteins/genes, metabolites and drugs of interest. IIS works in four connected modules: (i) Submission module, which receives raw data derived from Sanger sequencing (e.g. two-hybrid system); (ii) Search module, which enables the user to search for the processed reads to be assembled into contigs/singlets, or for lists of proteins/genes, metabolites and drugs of interest, and add them to the project; (iii) Annotation module, which assigns annotations from several databases for the contigs/singlets or lists of proteins/genes, generating tables with automatic annotation that can be manually curated; and (iv) Interactome module, which maps the contigs/singlets or the uploaded lists to entries in our integrated database, building networks that gather novel identified interactions, protein and metabolite expression/concentration levels, subcellular localization and computed topological metrics, GO biological processes and KEGG pathways enrichment. This module generates a XGMML file that can be imported into Cytoscape or be visualized directly on the web.

**Conclusions:**

We have developed IIS by the integration of diverse databases following the need of appropriate tools for a systematic analysis of physical, genetic and chemical-genetic interactions. IIS was validated with yeast two-hybrid, proteomics and metabolomics datasets, but it is also extendable to other datasets. IIS is freely available online at: http://www.lge.ibi.unicamp.br/lnbio/IIS/.

## Introduction

High-throughput screening of physical, genetic and chemical-genetic interactions brings new important perspectives in the Systems Biology field, as the analysis of these interactions provides new insights into protein/gene function, help to unravel how cellular networks are organized and facilitates the validation of therapeutic targets and drug design.

Recently, many experimental procedures have been developed to help elucidate the intricate networks of proteins, genes and drugs interactions, ranging from high-throughput experiments based on genomic scale analyses [1]–[6] to molecular biology approaches on a specific key pathway [7], [8]. Molecular interactions data related to human and model organisms are currently being integrated in diverse databases, such as BioGRID [9], Intact [10], DIP [11], STRING [12], MINT [13], HPRD [14], DrugBank [15], ChemBL [16], HMDB [17], YMDB [18], ECMDB [19], as well as KEGG [20] and Reactome [21]. However, the integration of different datasets is not a trivial task, since they vary widely in coverage, data quality and annotation. Moreover, the information available can be derived from diverse experimental methods, such as yeast two-hybrid (Y2H), mass spectrometry (MS), immunoprecipitation (IP), or fluorescence resonance energy transfer (FRET) assays to demonstrate protein interactions and, in some cases, interaction networks are determined solely by bioinformatics tools [22], [23], which rarely consider the subcellular localization of the interactors.

A major fraction of protein-protein interactions (PPIs) deposited in these public databases is generated by the yeast two-hybrid technology. Indeed, Y2H allows high-throughput screening of direct physical PPIs at a proteome scale, but requires the sequencing of hundreds to thousands of cellular preys per experiment. Moreover, the analyses of sequences derived from such interaction assays are difficult to proceed without an appropriate pipeline connecting different tools that can automatically integrate data derived from diverse sources and result in a more comprehensive and organized dataset that can be properly visualized and interpreted.

In response, several software projects became available to offer computer-assisted data and software integration. Notable among these are G2N [24], GeneMANIA [25], STRING [12], Ingenuity [26], and pISTiL [27] softwares. Though, most of them show some limitations. pISTil works well on chromatograms processing and partial annotation, but lacks the connection to visualization and analysis of interaction networks. The other software work well on the integration of a variety of bioinformatic tools with focus on the interaction networks, but lack the chromatograms processing feature or are restricted to a small number of model organisms and types of molecules.

Here we present the Integrated Interactome System (IIS), a new platform integrating a variety of tools and data sources used in systems biology analyses. It comprises a pipeline that receives raw sequence data from screening methods based on Sanger sequencing, like yeast two-hybrid system, or lists of proteins/genes, metabolites and drugs of interest, which are automatically processed, annotated and linked to interaction networks that can be filtered by the scoring system proposed by mathematical approaches, and evaluated according to expression/concentration fold change values and to the enriched biological processes and pathways in the network.

As major advantages over other systems, IIS supports the entire data analysis of experiments such as two-hybrid assays, besides other omics approaches, from the sequencing all the way to generating publication-ready interaction networks and annotation tables. In the process, all the challenges related to this type of experiment are addressed: processing/assembling reads, mapping them to the correct gene, automatically retrieving annotations from multiple resources and interactors from nine public databases, assigning annotations and interactions via orthologs if required, and building networks that gather novel identified interactions, protein and metabolite expression/concentration levels, subcellular localization, topological metrics and enriched biological processes and pathways. Each one of those tasks being very time-consuming and hard to manually integrate using separate different tools.

We also describe the construction of the Global Protein-Metabolite-Gene-Drug Interaction Database (GPMGDID) and discuss the workflow of IIS website. We then validate IIS's ability to perform the proposed tasks with three case studies: (i) human Nek6 yeast two-hybrid screening [28], (ii) *Saccharomyces cerevisae* encapsulated cells proteome [29] and (iii) primary and metastatic human ovarian cancer metabolome [30], on which we evaluate the benefits of using IIS to interpret the interaction profiles of a variety of conditions (e.g. interactions of specific genes or based on the omics data from different cell types or treatments).

## Methods

The Integrated Interactome System (IIS) is an integrative platform with a web-based interface, which integrates four different modules for processing, annotation, analysis and visualization of the interaction profiles of proteins/genes, metabolites and/or drugs of interest. IIS organizes the analysis in a project context and the user can create several projects protected by password. The project is a structure inside the system where researchers can develop and organize their thematic studies, choosing between two types: (i) chromatogram project or (ii) genes/metabolites/drugs project.

### Submission Module

The submission module is divided in nomenclature edition and chromatogram submission. The nomenclature edition allows the user to manage the description of the experiment, considering the laboratory, organism, cDNA library, strategy, project, sequencing plate and sequencing orientation. The chromatogram submission was developed to input the chromatograms (originated from Sanger sequencing derived from Y2H experiments, transcriptome, etc.) into the system. The chromatograms need to be organized in ZIP files and named according to the position in the 96 well plates used in the sequencing process (e.g. A01 to H12). The system receives the uploaded chromatograms file in a ZIP format (each file containing up to 96 chromatograms), checks the ZIP file and the individual chromatograms integrity after uncompressing, organizes the uncompressed chromatograms in a directory structure and runs PHRED base calling and quality scoring [31], generating reads sequences (FASTA and QUAL files) (Figure 1.1). The reads are then submitted to quality analysis and identification of vector and adaptor sequences, using the BDTrimmer program [32], and a report is sent by email to the user summarizing the information about the chromatograms processing (see also Methods S1 for more details). At the end of this module, the resulting processed reads are then aligned against a protein sequence database (GenBank/NR) by using the BLASTx alignment tool [33] with e-value threshold of 1e-10 to partially annotate them.

**Figure 1:**
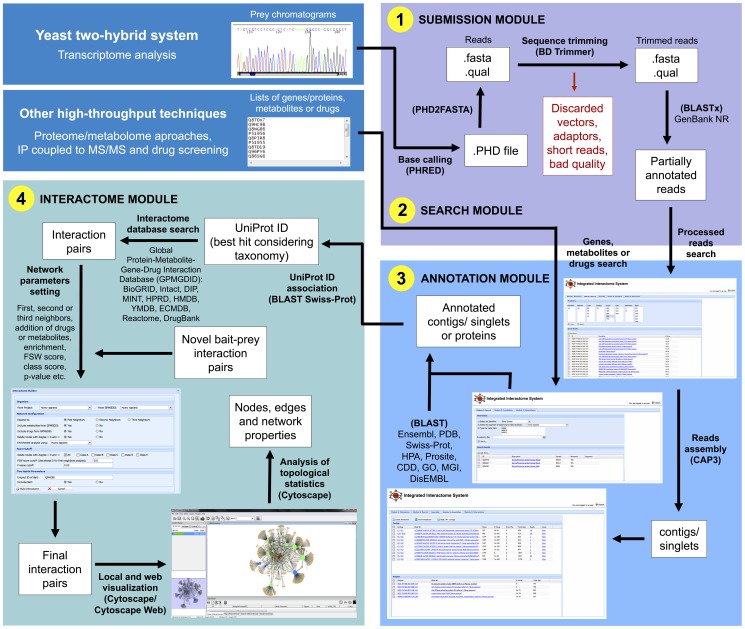
Workflow used in IIS, showing the integration of the (1) SUBMISSION, (2) ANNOTATION, (3) SEARCH and (4) INTERACTOME MODULES for data analysis. All steps are indicated by arrows alongside a term, out or in parentheses (both in black and bold font) that correspond to a sequence of actions (the term in parentheses meaning the tool/database used in that step).

### Search Module

In the second module, the partially annotated reads from the SUBMISSION MODULE are available to be checked, added to the user's project (chromatogram project type) and assembled into clusters (contigs and singlets) using CAP3 program [34], in order to eliminate redundant reads typically generated by Y2H and transcriptome assays (Figure 1.2).

In the genes/metabolites/drugs project type, lists of genes/proteins (UniProt Accession, RefSeq or gene symbol), metabolites (HMDB, YMDB or ECMD IDs) and/or drugs (DrugBank ID or CAS number) can also be uploaded by the user as a single column TXT file and added to the project (Figure 1.2). Because of the gene symbols redundancies and the presence of aliases in the databases, searching for gene symbols in the selected organism is first performed on Swiss-Prot database and in the case of unreviewed proteins it is extended to TrEMBL database. It is also possible to upload a two-column TXT file containing UniProt Accession, RefSeq or gene symbol and fold change values, respectively, the second one representing expression/concentration levels.

### Annotation Module

In the third module, the partially annotated contigs and singlets, or the lists of proteins/genes uploaded by the user, are searched against nine databases (Gene Ontology [35], HPA [36], CDD [37], MGI [38], PDB [39], DisEMBL [40], Prosite [41], Ensembl [42] and Swiss-Prot [43], all of them queried monthly for updates) in order to generate tables with automatic annotation that can be exported to other software (e.g. Excel) for editing/formatting purposes (Figure 1.3). The lists of proteins/genes are searched by their respective UniProt Accession numbers, and the contigs/singlets are first blasted against Swiss-Prot database and their best hits used to make an association between their sequences from the selected organism defined by the user and their respective UniProt Accession numbers [44].

This module was designed in order to allow users to export publication-ready tables with a more complete annotation of their data (for an example and more details see Table S1 and Methods S1). Users can also create their annotation tables containing only some desired fields by selecting the databases of interest. By doing this, thematic annotation tables can be created, e.g. structural annotation tables (by selecting the CDD, PDB, DisEMBL and Prosite databases), functional annotation tables (by selecting the Gene Ontology, HPA and MGI databases), etc., according to the user needs.

### Interactome Module

The fourth module is used to blast the input contigs/singlets against the Swiss-Prot database to retrieve the corresponding UniProt Accession numbers of the organism of interest, or search the input lists of proteins/genes, metabolites and drugs that are already linked to their own or related UniProt Accession numbers, and use them as queries in our Global Protein-Metabolite-Gene-Drug Interaction Database (GPMGDID) to build the networks (Figure 1.4). The latter was constructed in a MySQL structure by grouping more than 1 million interactions from nine public available databases: BioGRID [9], Intact [10], DIP [11], MINT [13], HPRD [14], DrugBank [15], HMDB [17], YMDB [18], and ECMDB [19], all of them queried monthly for updates. There are five parameters classes to select in this module: the organism, the network configuration, the score cutoff, the two-hybrid parameters and the expression analysis. IIS works with diverse organism datasets that can be chosen independently for the input dataset (project) and the GPMGDID, enabling also the construction of networks with interactions between different organisms (e.g. host-pathogen interactions) or using ortholog relationship. The network configuration parameter considers the interaction level of expansion from first to third neighbors, the addition or not of metabolites and drugs from GPMGDID in the network expansion, the deletion of nodes with connectivity degree of 0 and 1 (yielding a more connected network), and the selection of the background organism for the enrichment analysis. The score cutoff parameters can be used to filter the network for more confident interactions by three types of score: the Class score, the FSW score and the p-value, which are described in more details in the following sections. The order considered in the algorithm to reduce the network size by filters is: (i) Class score, (ii) p-value, (iii) deletion of nodes with connectivity degree of 0 and 1, and (iv) FSW score. In the two-hybrid parameters, if the user is working with two-hybrid or immunoprecipitation techniques and has a bait of interest to connect with the identified novel preys, it can be done using this option. Finally, in the expression analysis parameters, if working with omics datasets, the user can set cutoff values to color the input nodes as up- or down-regulated and change the node sizes according to their fold change in expression/concentration levels. Regarding the enrichment analysis, the program calculates the enrichment for the GO biological processes and KEGG pathways in the generated network using the hypergeometric distribution [45]. The exact and approximated hypergeometric distributions were implemented in the interactome algorithm using gamma and log-gamma function, respectively, to calculate factorial number. The second one was necessary to avoid stack overflow related to large factorial numbers [46] (the empirical tests showed that the transition from exact to approximated function occurs for GO term or KEGG pathway with more than 1,800 related proteins in the GPMGDID database).

This module generates a XGMML file containing all annotations and metrics described below that can be directly visualized on the website using Cytoscape web [47] from our web server (Figure 1.4) or can be imported into Cytoscape platform [48]. The Cytoscape platform is an open source software that enables the visualization of all interactions (or defined subgroups of interactions) and the analysis and correlation of node and edge properties with topological network statistics using a set of core modules and external plugins. The information available in the XGMML file has been standardized in order to communicate with these plugins.

#### Construction of the GPMGDID database

The Global Protein-Metabolite-Gene-Drug Interaction Database (GPMGDID) is a non-redundant database which integrates all protein-metabolite-gene-drug interactions described in several public databases, divided by organism, where the interaction pairs are classified by data type (experimental or predicted), methodology (e.g. two-hybrid, pull down, genetic interference, etc.), organism and source (PubMed ID of the paper that published the interaction), while the proteins/genes involved in the interactions are characterized by biological process, molecular function and cellular component allowing the enrichment and compartmentalization analysis performed by the INTERACTOME MODULE.

**Figure 2:**
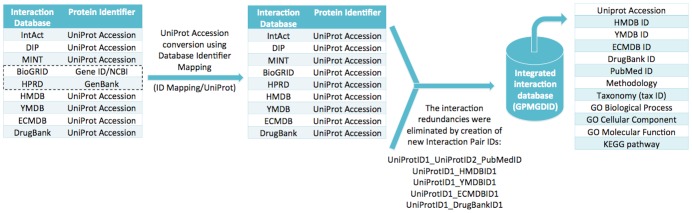
Global Protein-Metabolite-Gene-Drug Interaction Database (GPMGDID) construction. The UniProt Accession was chosen as the reference ID for the unification of the nine different databases used to construct GPMGDID: IntAct, DIP, MINT, BioGRID, HPRD, HMDB, YMDB, ECMDB and DrugBank databases. The interaction redundancies were eliminated by concatenating pairs of interactions with the source (PubMed IDs), generating an interaction pair ID given by UniProtID1_UniProtID2_PubMedID. The resultant database integrates several protein-metabolite-gene-drug interactions classified by source, methodology and organism.

The publicly available interaction databases have non-standard protein identifications, file formats and are not uniquely indexed and annotated, which compromises the development of a single algorithm to integrate all datasets. Therefore, the UniProt Accession was chosen as the reference ID for the unification of the different datasets, generating the following possible interaction pairs: UniProtID1_UniProtID2, UniProtID1_HMDBID1, UniProtID1_YMDBID1, UniProtID1_ECMDBID1 or UniProtID1_DrugBankID1. A large amount of interaction redundancies generated because the same information is described in different interaction databases was also eliminated by concatenating interaction pairs with the source from which they were described (PubMed IDs), producing an interaction pair ID given by UniProtID1_UniProtID2_PubMedID. Figure 2 summarizes the pipeline applied to construct GPMGDID database (see also Methods S1 for more details).

#### Filtering high-confidence interactions by mathematical approaches

The interacting pairs constructed by the method described above may be error prone and must undergo a validation step. In order to achieve a more reliable result, some facts should be considered: proteins that actually interact are expected to share the same cellular compartment and have common interaction partners. It has been shown that a pair of genuine interacting proteins is generally expected to have a common cellular role and proteins that have common interaction partners have a higher chance of sharing a common function. Moreover, even if two proteins are consistently predicted to interact they must be located at the same cell compartment and at the same time [49]–[53]. Therefore, three validation approaches were considered to verify the quality of interaction pairs in networks constructed from GPMGDID database: Class score, Functional Similarity Weight score (FSW score) and p-value. These mathematical approaches are further described and can be used as filters in the INTERACTOME MODULE to reduce the network size for more reliable interactions.

#### Class score

The interactions in the GPMGDID present a Class score similar to the cellular compartment classification (C^3^) described by Brandão et al. [54], and it is based on three characteristics: type of interaction (experimental or predicted), number of papers describing the interaction in PubMed (PubMed ID), and cellular component (CC) described for the interacting nodes in the Gene Ontology database [35]. The CC used by IIS corresponds to a concise list of the main selected subcellular compartments from GO and are depicted in bold in Table S2 (GO CC children terms were grouped for each selected main ancestral CC term, considering only terms annotated for ≥10 genes). This classification divides the interactions into four classes according to their evidence and subcellular localization. Class score value attributed for the type of interaction is +4 if it is based on experimental data, and 0 if there is no experimental data available (predicted); for co-localization we attribute score +1, otherwise we display score 0; if the interaction is described in more than one PubMed ID considering at least one paper not related to high-throughput experiments we score +4, if the interaction is described in more than one PubMed ID we score +3, if it is described in only one PubMed ID we score +1, and 0 if not published. We consider high-throughput experiment papers those describing more than 500 interactions. The Class scores are used in IIS to depict different edge widths to the generated networks, in order to visually assign interactions confidence (Table 1).

**Table 1:** Interactions confidence measured by Class scores used to represent different edge widths in the networks.

Class	Score	Edge width	Parameters^1^
A	+9	2.5	Experimental/PubMed ID >1 (at least one not HT)/same CC
B	+7/+8	2.0	Experimental/PubMed ID >1 or Experimental/PubMed ID >1/same CC
C	+6	1.5	Experimental/PubMed ID = 1/same CC
D	+5	1.0	Experimental/PubMed ID = 1
E	+4	0.5	Experimental/PubMed ID = 0

1 Parameters used to calculate the Class scores: interaction described as experimental (not predicted) (+4); interaction described in more than one paper (PubMed ID >1) and at least one paper not describing high-throughput (HT) experiments (+4); interaction described in more than one paper (PubMed ID >1) (+3); interaction described in only one paper (PubMed ID  = 1) (+1); interacting nodes described in the same cellular component (CC) (+1). For novel interactions not described in any paper (PubMed ID  = 0), even if the interacting nodes are described in the same CC, it will be assigned Class score E.

#### Functional Similarity Weight score (FSW score)

In GPMGDID, due to its integrative profile, the reliability index for a reported interaction can be postulated in terms of the proportion of interaction partners that two proteins have in common. A mathematical approach called Functional Similarity Weight (FSWeight) [51] has been proposed to assess the reliability of protein interaction data based on the number of common neighbors of two proteins. The FSWeight approach was initially designed to predict protein functions, and lately has shown a good performance in evaluating the reliability of protein interactions [53]. The interaction pairs of proteins that are classified with high score by this method are likely to be true positives. On the other hand, the pairs of proteins that are classified with low scores are likely to be false positives. The most interesting feature of the FSWeight is that it is able to rank the reliability of an interaction between a pair of proteins using only the topology of the interactions between that pair of proteins and their neighbors within a short radius in a graph network [49], [50].

Therefore, we implemented in GPMGDID the Functional Similarity Weight score (FSW score) calculation originally proposed by Chua et al. [51], and described by Brandão et al. [54], for all first, second and third level interactions present in our database. The effect of FSW score threshold in the network is exemplified and discussed in the Results and Discussion section.

#### P-value

Finally, a statistical hypothesis testing was implemented to avoid random interaction pairs generated during network expansion using GPMGDID database. Every time the user builds a new subnetwork from the GPMGDID, p-values are calculated for each protein in the generated subnetwork, in order to assign confidence. The p-value is calculated based on the work by Berger et al. [24]. First, the z-score value is calculated for each protein using a binomial proportion test that depends on the total of interactions of the protein in the subnetwork, the total of interactions of the protein in the GPMGDID filtered by a specific organism, the total of interaction pairs in the subnetwork and the total of interaction pairs in the GPMGDID filtered by a specific organism. Next, a normal distribution that depends on the variance and average of the values already calculated was used for converting the z-scores to p-values.

#### Web interface

IIS web interface was built in JavaScript, JSON and PHP, and locally hosted on a Linux server at http://www.lge.ibi.unicamp.br/lnbio/IIS/. The web interface allows the user to work in the thematic project, protected by password, organizing and updating the set of proteins/genes, metabolites and drugs of interest and their respective annotations and networks.

## Results and Discussion

We have validated IIS's ability to perform the analysis of interaction profiles for both specific genes or omics data originated from different cell types or conditions with three case studies: (i) an yeast two-hybrid screening [28], (ii) an yeast proteome [29] and (iii) a human cancer metabolome [30].

### First case study: hNek6 yeast two-hybrid screening

**Figure 3:**
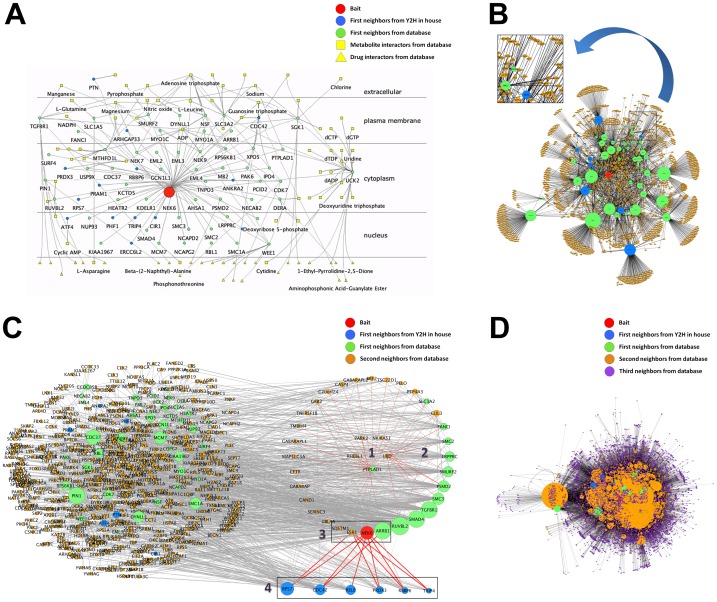
Human Nek6 interactome built from yeast two-hybrid data. (A) hNek6 first neighbors network, showing the bait hNek6 in red, the Y2H first neighbors in blue, the first neighbors described in the GPMGDID database in green, and the metabolites/drugs interactors described in the GPMGDID database in yellow and in different shapes: squares for metabolites and triangles for drugs. The proteins were localized according to their cellular components (GO) described in the “Selected CC” node attribute field by using the Cerebral Cytoscape plugin. (B) hNek6 second neighbors network, showing the second neighbors in orange. The proteins were distributed according to the organic layout. The insertion is depicting the different edge widths, according to our confidence Class scores. (C) hNek6 second neighbors network showing the following protein clusters: 1. top enriched NF-kappaB cascade, 2. first neighbors of cluster 1, 3. enriched NF-kappaB cascade subset of cluster 2, and 4. hNek6 yeast two-hybrid interactors. The proteins were distributed according to the organic and degree-sorted circle layouts, and proteins with degree 0 and 1 were deleted from the network. (D) hNek6 third neighbors network, showing the expansion from the first to the third level of interaction with the third neighbors in purple. The proteins were distributed according to the organic layout. The networks were visualized using Cytoscape v2.8.3.

The human NIMA-related kinase 6 (hNek6) was chosen based on a previous work by our group [28] in which the PPI network of hNek6 was manually generated, annotated and visually analyzed by Osprey software [55] using the BioGRID database [9]. Here we used IIS to perform all the steps from the chromatograms processing to the annotation and interactome construction and analysis using a standardized pipeline executed in a significantly shorter period of time. First, hNek6 prey cDNAs were sequenced and their chromatograms were organized into files to be submitted to IIS in a new chromatogram project. After submission, the chromatograms were immediately processed into reads, assembled into contigs and singlets and blasted against GenBank/NR for a partial annotation. The complete annotation table (Table S1) against diverse databases was then generated by selecting all the contigs and singlets in the “Module 3: Annotation” tab inside the project and using the “Create Annotation” button. The same selection was done in the “Module 4: Interactome” tab, and the “Create Interactome” button was used to build the hNek6 networks, from the first to the third neighbors levels of interactions (Figure 3A, B and D). All the networks were visualized both on the website using the Cytoscape web [47], and locally using the Cytoscape software [48], which was also used to manipulate and analyze the networks. The example chromatogram files available for the user on the IIS website correspond to fifteen hNek6 interactions confirmed by *in vitro* and *in vivo* assays (described in Table 5 by Meirelles et al. [28]).

Figure 3 shows the hNek6 interaction networks generated by IIS and visualized by Cytoscape. Our new automatic analysis using IIS made it possible to verify, as described before [28] that this kinase is a hub (node with several connections in the network) involved in several biological processes through its interaction with diverse types of proteins in different cellular compartments (Table S3), possibly at different time points during the cell cycle. In our previous work [28], we manually curate from the literature the hNek6 putative cellular roles, considering all the novel interacting partners retrieved by the yeast two-hybrid screening, which were as follows: cell cycle, cytoskeleton organization, DNA repair, NF-kappaB and Notch signalings and cancer-related interactions. Using our new approach, by building a network for the hNek6 interactions confirmed by *in vitro* and *in vivo* assays, considering only the top enriched biological processes (p≤0.05) and, particularly, the second neighbors expansion, we were able to identify mostly the same processes but also new ones, e.g. apoptotic process (GO enrichment p-value of 2.2e-48), cell division (1.1e-41), epidermal growth factor receptor signaling pathway (3.3e-38), transcription, DNA-dependent (6.8e-34), cell proliferation (1.0e-31), DNA repair (1.5e-22), I-kappaB kinase/NF-kappaB cascade (1.7e-16) and others (Table S3). Beyond cell cycle and DNA repair biological processes/pathways, which have been more extensively explored for Neks [56], the NF-kappaB cascade kept our attention, since NEK6 gene was described among others to activate the NF-kappaB signaling pathway, in a large-scale screening [57]. However, there is no explanation of how hNek6 activates this pathway and the first possible links to that question were addressed by our yeast two-hybrid results that showed hNek6 interactions with Transcription factor RelB (RELB), Prx-III (PRDX3) and TRIP-4 (TRIP4) [28]. The first neighbors expansion of our network was not able to show enrichment in I-kappaB kinase/NF-kappaB cascade, but in apoptotic process and transcription, where these proteins were found as most enriched. Though, from the second neighbors expansion, we could observe I-kappaB kinase/NF-kappaB cascade enrichment, forming a cluster of five proteins: Protein-tyrosine phosphatase-like A domain-containing protein 1 (PTPLAD1), NF-kappa-B inhibitor-interacting Ras-like protein 1 (NKIRAS1), E3 ubiquitin-protein ligase parkin (PARK2), GTPase RhebL1 (RHEBL1) and Ubiquitin D (UBD) (Figure 3C). Interestingly, the first neighbors of this cluster have hNek6 as a component forming another smaller cluster of proteins also annotated to be involved in NF-kappaB cascade (by analyzing all their enriched biological processes depicted in Table S3): hNek6 (NEK6), Beta-arrestin-1 (ARRB1), Estrogen receptor (ESR1) and Sequestosome-1 (SQSTM1). Moreover, five hNek6 protein partners identified by the yeast two-hybrid system (40S ribosomal protein S7, Cell division control protein 42 homolog, E3 ubiquitin-protein ligase RBBP6, Prx-III and TRIP-4), including two of the three interactors described above also interact with two other proteins from this hNek6 cluster (Beta-arrestin-1 and Estrogen receptor), both of which negatively regulate NF-kappaB cascade [58], [59] (Figure 3C, red edges). Therefore, our hypothesis is that hNek6 may interact directly with any of those two-hybrid interactors, possibly regulating them by phosphorylation, which could regulate their interaction with Beta-arrestin-1 and/or Estrogen receptor, finally inhibiting these proteins and activating the pathway. This analysis adds novel possible clues on how hNek6 activates NF-kappaB cascade. Although the Transcription factor RelB was found to interact only with hNek6 from the referred cluster, it is already a direct link to the NF-kappaB cascade activation, since it is a component of the NF-kappa-B RelB-p50/p52 complex. Nek6 is also directly linked to Protein-tyrosine phosphatase-like A domain-containing protein 1 (PTPLAD1), enriched in the I-kappaB kinase/NF-kappaB cascade cluster. Altogether, these findings may suggest a novel non-mitotic function for hNek6 through this pathway.

### Second case study: *S. cerevisiae* encapsulated cells proteome

As an example of a proteomics study, we chose the *S. cerevisiae* proteome of encapsulated cells in liquid core alginate-chitosan capsules in comparison with cells grown freely in suspension described by Westman et al. [29]. In the context of bioethanol production, encapsulation of yeast cells has been shown to improve the fermentative performance in toxic lignocellulosic hydrolysates [60] and to increase thermotolerance [61]. It has been shown that the yeast metabolism changed significantly upon encapsulation [29], so we used IIS to build a network for the 116 up- and 95 down-regulated proteins in yeasts growing in capsules (described in Table S1 by Westman et al. [29]) to comparatively analyze how encapsulation affects the cells on a more integrated molecular level. First, we uploaded a single two-column TXT file containing both the up- and down-regulated proteins, available as UniProt Accession numbers and respective fold change values, in the “Module 2: Search” tab inside the project. Then the retrieved proteins were selected and added to the project, annotated in the “Module 3: Annotation” tab, and used as queries to build a network in the “Module 4: Interactome” tab, setting expression analysis parameters to consider fold change ≥1.3 as up-regulated and fold change ≤−1.3 as down-regulated proteins. The network was visualized and manipulated using the Cytoscape software.

**Figure 4:**
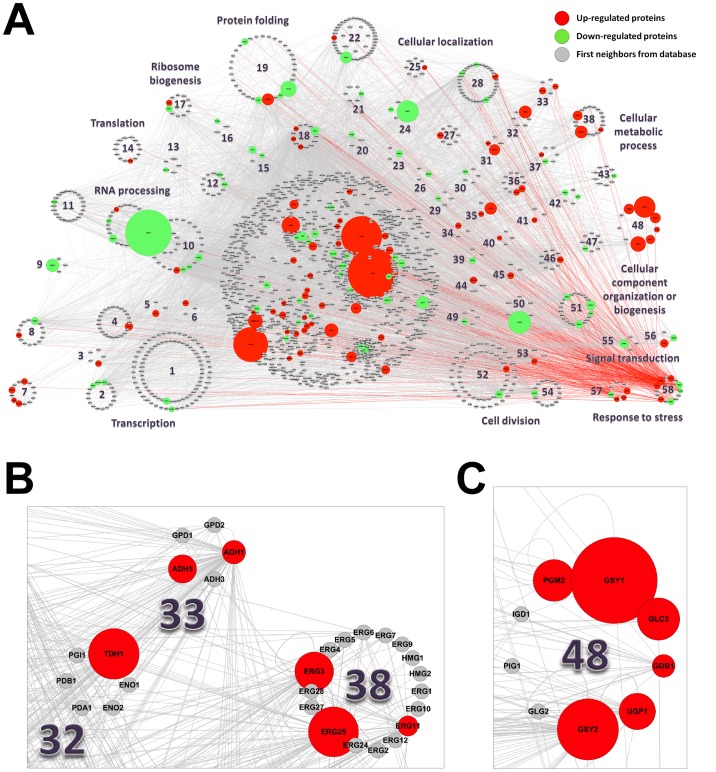
Interactome of *S. cerevisiae* encapsulated in liquid core alginate-chitosan capsules vs. cells grown freely in suspension, built from proteome data. (A) The enriched GO biological processes (p≤0.05) among the up-regulated proteins (red), the down-regulated proteins (green) and the background intermediary proteins (grey) from GPMGDID are depicted in the network by clustering the proteins involved in each of the biological processes with a circle layout. Clusters were assigned only to biological processes containing more than three proteins with at least one from the proteome data; proteins belonging to more than one biological process were assigned to clusters with the best enrichment p-values. More specific biological processes are shown only for proteins with more specific annotation in GO database. The nodes sizes of up- and down-regulated proteins are depicted proportional to their fold change (FC ≥1.3, FDR p≤0.05, as described by Westman et al.) [29]. (B) Network zoom showing the glycolysis (GO enrichment p-value of 1.7e-02), NADH oxidation (2.1e-04) and ergosterol biosynthetic process (4.3e-15) clusters. (C) Network zoom showing the glycogen biosynthetic process (2.5e-06) cluster. The network was built using first neighbors expansion, deletion of nodes with degree 0 and 1, addition of different colors and sizes to proteins according to their fold change, and was filtered by Class scores A to C. The network was visualized using Cytoscape v2.8.3 and the proteins were distributed according to selected enriched biological processes (GO) from the “Top Enriched BP” node attribute field by using the group attributes layout. The following enriched biological processes clusters are shown in the network: 1. transcription, DNA-dependent (3.8e-25), 2. chromatin silencing at telomere (6.1e-15), 3. positive regulation of RNA elongation from RNA polymerase II promoter (5.0e-10), 4.positive regulation of transcription from RNA polymerase II promoter (4.2e-19), 5. negative regulation of transcription, DNA-dependent (7.1e-03), 6. positive regulation of transcriptional preinitiation complex assembly (2.5e-05), 7. vacuolar acidification (1.1e-10), 8. replicative cell aging (3.9e-12), 9. pseudohyphal growth (3.6e-08), 10. rRNA processing (4.8e-16), 11. maturation of SSU-rRNA from tricistronic rRNA transcript (1.2e-15), 12. regulation of translation (3.2e-10), 13. regulation of translational fidelity (2.7e-05), 14. mitochondrial translation (9.9e-04), 15. mature ribosome assembly (5.2e-04), 16. ribosomal small subunit assembly and maintenance (1.1e-05), 17. ribosomal large subunit biogenesis and assembly (3.9e-12), 18. protein refolding (1.8e-11), 19. protein folding (7.7e-09), 20. mRNA transport (9.7e-08), 21. poly(A)+ mRNA export from nucleus (5.2e-09), 22. protein transport (1.9e-11), 23. ribosomal small subunit export from nucleus (1.3e-08), 24. protein localization (4.9e-07), 25. protein import into nucleus (6.9e-11), 26. protein targeting to ER (5.2e-04), 27. ER to Golgi vesicle-mediated transport (1.1e-07), 28. endocytosis (9.6e-19), 29. lysine biosynthetic process via aminoadipic acid (7.1e-03), 30. pantothenate biosynthetic process (4.5e-04), 31. heme biosynthetic process (1.3e-03), 32. glycolysis (1.7e-02), 33. NADH oxidation (2.1e-04), 34. phospholipid biosynthetic process (1.1e-02), 35. fatty acid metabolic process (8.9e-04), 36. fatty acid biosynthetic process (2.7e-05), 37. protein amino acid N-linked glycosylation (2.1e-03), 38. ergosterol biosynthetic process (4.3e-15), 39. branched chain family amino acid catabolic process (2.4e-04), 40. pentose-phosphate shunt (2.7e-02), 41. 2-oxoglutarate metabolic process (2.4e-03), 42. one-carbon compound metabolic process (1.2e-02), 43. DNA recombination (5.8e-03), 44. metabolic process (3.0e-03), 45. deoxyribonucleotide biosynthetic process (6.6e-06), 46. protein deubiquitination (1.8e-09), 47. aerobic respiration (3.3e-04), 48. glycogen biosynthetic process (2.5e-06), 49. actin cytoskeleton organization and biogenesis (3.0e-05), 50. actin filament organization (5.1e-12), 51. chitin- and beta-glucan-containing cell wall organization and biogenesis (1.0e-12), 52. cell division (2.8e-21), 53. mitosis (1.5e-17), 54. establishment of cell polarity (2.8e-13), 55. TOR signaling pathway (8.3e-09), 56. Ras protein signal transduction (1.2e-07), 57. response to osmotic stress (1.1e-10), 58. response to stress (3.3e-06).

Figure 4 shows the interactome of encapsulated *S. cerevisiae* built from the proteome data. Our new analysis using IIS showed the same and other functional categories enriched among the up- and down-regulated proteins as described before [29], but using the GO database instead and with one considerable advantage: together with Cytoscape it enabled the visualization of the (i) distribution of the biological processes among the identified proteins, (ii) the number, identity and type of each protein (up- or down-regulated and interactors from database) in each process, (iii) the relative fold change levels of each protein and (iv) their interactions, all resultant data integrated in the same network. It was also possible to analyze the network according to the enriched KEGG pathways and GO cellular components, since these information were also computed and available in the generated network (data not shown).

In a more global perspective, it was of immediate observation that the majority of up-regulated proteins was involved in cellular metabolic processes (eg. heme biosynthetic process, glycolysis, NADH oxidation, fatty acid metabolic process, ergosterol biosynthetic process and glycogen biosynthetic process), unlike the down-regulated proteins, mostly involved in RNA processing (comprising the most down-regulated protein Drs1p), translation and cellular component organization or biogenesis (Figure 4A). Regarding the metabolic process clusters in the network, as also emphasized by Westman et al. [29], the glycolytic pathway enzyme Tdh1p was found in a significantly higher level in the encapsulated yeast (Figure 4B), and the high affinity hexose transporters Hxt6p and Hxt7p, although not clustered together, were visually identified as the most up-regulated proteins. Moreover, our analysis was able to identify many proteins in the glycogen biosynthetic process cluster (eg. Gsy1p, Gsy2p, Pgm2p, Glc3p, Ugp1p and Gdb1p) (Figure 4C), and proteins involved in NADH oxidation (the alcohol dehydrogenases Adh1p and Adh5p, which reduce acetaldehyde to ethanol) (Figure 4B), which were all up-regulated. These findings strongly indicate a carbon limitation inside the capsules, but an accumulation of glycogen as the capsules filled up with cells, considering its importance as a storage carbohydrate in slowly growing or starved yeast, and, more relevant, an increase in ethanol yields. Notably, proteins involved in the ergosterol biosynthetic process cluster (eg. Erg25p, Erg3p and Erg11p) were also visually identified as greatly up-regulated (Figure 4B), although not discussed in the previous report by Westman et al. [29]. Since ergosterol is the major sterol of the fungal plasma membrane, important for the fluidity and integrity of the membrane and for the proper function of many membrane-bound enzymes, with its biosynthetic pathway consisting in a pivotal target of antifungal drugs [62], these findings may also explain the differences between encapsulated and free growing yeast cells. Indeed, a more intact membrane supports higher concentrations of ethanol. Furthermore, among the stress response proteins, comprising both up- and down-regulated proteins, it was suggested by Westman et al. [29] that a more plausible explanation for the apparent osmotic stress response is a cross-talk between nutrient starvation and other environmental stress responses. In our network analysis, this hypothesis could be visualized by the broad spectrum of connections among the stress response clusters with other clusters in the network (Figure 4A, red edges).

### Third case study: primary and metastatic human ovarian cancer metabolome

For the metabolomics analysis, we used as an example the work by Fong et al. [30], which described the metabolome of the human normal ovary and its transformation in primary epithelial ovarian cancer (EOC) and metastatic ovarian cancer (MOC). In the context of oncogenesis and the importance of a comprehensive metabolic analysis of solid tumors to reveal possible biomarkers for early diagnosis and monitoring of cancer progression and recurrence, IIS was used to build two comparative networks: one for the up- and down-regulated metabolites in EOC and the other one for the up- and down-regulated metabolites in MOC (described in Table S2 by Fong et al. [30]). First, we converted the metabolite names to HMDB IDs and uploaded a single two-column TXT file containing both the up- and down-regulated metabolites for each condition (EOC and MOC), as a list of HMDB IDs and respective fold change values, in the “Module 2: Search” tab inside each project (EOC and MOC). Then the retrieved metabolites were selected and added to the project, and used as queries to build the networks in the “Module 4: Interactome” tab, setting expression analysis parameters to consider fold change ≥1.2 as up-regulated and fold change ≤−1.2 as down-regulated metabolites, as described by Fong et al. [30]. The network was visualized and manipulated using the Cytoscape software.

**Figure 5:**
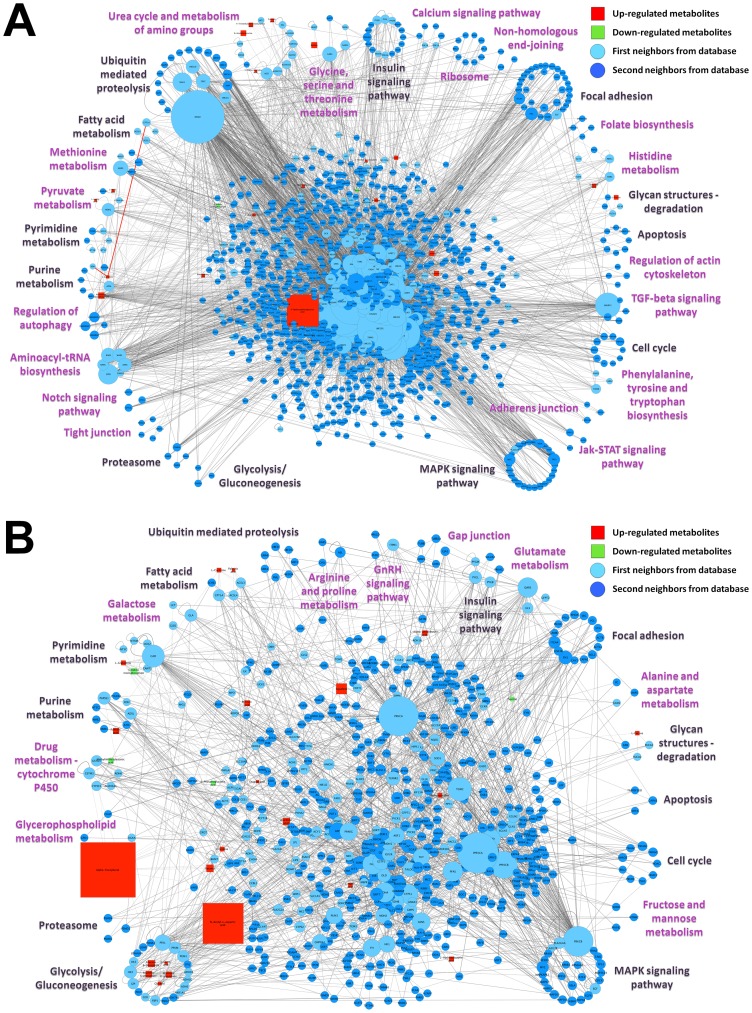
Comparison between the interactomes of (A) primary human epithelial ovarian cancer and (B) metastatic ovarian cancer vs. normal human ovary, built from metabolome data. The enriched KEGG pathways (p≤0.05) among the up-regulated metabolites (red squares), the down-regulated metabolites (green squares) and the background intermediary proteins (light blue circles for first neighbors and dark blue circles for second neighbors) from GPMGDID are depicted in the networks by clustering the proteins involved in each of the pathways with a circle layout. Enriched KEGG pathways specifically for each network (A) or (B) are depicted in purple and the ones in common are depicted in black. Clusters were assigned only to pathways containing more than three proteins (disease pathways or pathways specific for defined cell types were not considered), and metabolites were assigned only to metabolic pathway clusters containing interacting proteins with the best enrichment p-values. The nodes sizes of up- and down-regulated metabolites are depicted proportional to their fold change (FC≥1.2, p≤0.05, as described by Fong et al. [30]) and the nodes sizes of the background intermediary proteins are depicted proportional to their connectivity degree. The networks were built using second neighbors expansion, deletion of nodes with degree 0 and 1 and addition of different colors and sizes to proteins according to their fold change. The networks were visualized using Cytoscape v2.8.3 and the proteins were distributed according to selected enriched pathways (KEGG) from the “Top Enriched KEGG” node attribute field by using the group attributes layout.

Figure 5 shows the interactomes of (A) EOC and (B) MOC built from the metabolome data. Our new analysis using IIS showed similar metabolic pathways as described before [30], and also other signaling and metabolic pathways enriched among the up- and down-regulated metabolites. We analyzed the data based on the KEGG database enrichment performed by IIS, which was able to retrieve 5 of each list of 15 enriched pathways for EOC and MOC identified by the Ingenuity Pathway Analysis (IPA): Aminoacyl-tRNA biosynthesis (KEGG enrichment p-value of 1.3e-26), Urea cycle and metabolism of amino groups (1.4e-21), Glycine, serine and threonine metabolism (1.1e-10), Methionine metabolism (2.5e-09), and Phenylalanine, tyrosine and tryptophan biosynthesis (1.4e-07) for EOC; and Alanine and aspartate metabolism (1.8e-16), Purine metabolism (8.3e-12), Arginine and proline metabolism (2.3e-11), Glutamate metabolism (2.0e-10), and Pyrimidine metabolism (1.2e-10) for MOC. Though, IIS also retrieved other 25 and 16 significant enriched pathways for EOC and MOC, respectively, including signaling and metabolic pathways (Figure 5). Notable among these are the Glycan structures degradation (1.3e-04 in EOC; 3.0e-09 in MOC) and Fatty acid metabolism (1.3e-11 in EOC; 2.1e-14 in MOC) pathways enriched in both EOC and MOC, which could explain the increase in fucose (2.75 fold in EOC; 1.81 fold in MOC) and carnitine (1.79 fold in EOC; 1.88 fold in MOC) levels. The enriched Pyruvate metabolism (5.6e-17) and Glycolysis/Gluconeogenesis (5.5e-32) pathways in EOC and MOC, respectively, could also explain the increase in lactate levels when compared to normal ovarian tissue (1.46 fold in EOC; 1.37 fold in MOC).

In order to reduce complexity, Figure 5 shows the metabolites in only a few metabolic pathway clusters, since they are the ones containing interacting proteins with the best enrichment p-values, although the metabolites are also connected to the other clusters by interactions with different proteins, e.g. carnitine is connected to the Purine metabolism (7.9e-36) cluster in EOC by its interaction with Xanthine dehydrogenase/oxidase (XDH), and also connected to the Fatty acid metabolism (1.3e-11) cluster by its interaction with Carnitine palmitoyltransferase 1A (CPT1A) (Figure 5A, red edges). Clusters composed of at least one first neighbor interactor represent probably the most confident pathways, since they group direct interactors of metabolites. As in the proteomics approach, IIS metabolomics analysis connected to Cytoscape enabled the visualization of all resultant data integrated in the same network, making it easier to interpret the whole dataset and its relations, since they can bring together information concerning: the (i) distribution of the pathways among the identified metabolites, (ii) the number, identity and type of each metabolite (up- or down-regulated) in each process, (iii) the relative fold change levels of each metabolite and (iv) their interactions.

### Network attributes and parameters

It is important to point out that the network construction by IIS considers the degree of each node in the network, showing a gradient of node sizes, which makes it easy to distinguish the hubs. The generated network also brings the cellular components and the enriched biological processes and pathways of each node, which can be used to easily separate the nodes into cell compartments (e.g. by using different layouts or the Cerebral Cytoscape plugin [63], as shown in Figure 3A), or cluster the nodes into functional modules. It also considers each type of node (proteins, metabolites and drugs) as different entities, which can be distinguished by their different node shapes (Figure 3A), and depicts different edge widths according to the interaction confidence Class score, described above (Figure 3B). Other confidence interaction measures, such as the FSW score and p-value, or different types of interactions, can be accessed as node and edge attributes (Methods S1). Besides, all of these parameters can be changed by the users according to their specific needs.

**Figure 6:**
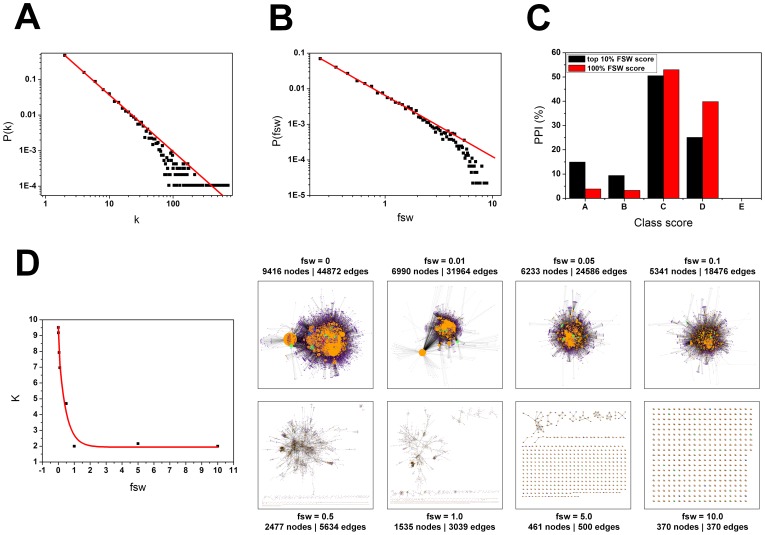
Comparison between FSW score, degree and Class score. (A) Degree distribution of hNek6 third neighbors network (γ = −1.59). (B) FSW score distribution of hNek6 third neighbors network (γ = −1.72). (C) Percentage of PPIs characterized by the best FSW score and Class score in hNek6 third neighbors network. (D) Correlation between the average degree and the FSW score of hNek6 third neighbors network from FSW score 0 to 10. Both the degree distribution and the FSW score distribution approximate a power-law and are scale-free in topology. The slopes (γ) were determined by linear fitting where *P*(*k*) approximates a power-law: *P*(*k*)≈*k* ^−*γ*^ (*k*: total number of links; K: average degree; γ: slope of the distribution on the log-log plot; fsw: functional similarity weight; PPI: protein-protein interaction).

As a metric of how many neighbors a pair of proteins share, the FSW score was implemented so that it can also be used as a filter of hubs when building the networks. To statistically compare the FSW distribution to the degree distribution in the networks generated by IIS, the degree distribution *P*(*k*) and the average degree (*K*) of each network were calculated as described by Stelzl et al. [4]. We found that the FSW score distribution is similar to the degree distribution and is also scale-free in topology (Figure 6A and B). Therefore, the FSW score could be used as a parameter to filter hubs from the networks, as shown by using score values 0.01 to 1.0, where the average degree of the network is greatly reduced and most of the hubs fall outside the network (Figure 6D).

Furthermore, the effectiveness of using FSW score as a PPI reliability index was demonstrated before [49], [50], [54]. Here we ranked the top 10% of protein interactions in the hNek6 third neighbors network by the FSW score and compared to the Class score. We found that the top 10% of PPIs with the best FSW scores were also enriched with the best Class scores A and B: 15.0% were characterized by Class score A and 9.4% by Class score B, compared to 3.8% and 3.3%, respectively, considering the total PPIs in the network (Figure 6C).

IIS annotates nodes and edges using diverse databases and metrics, and offers a variety of filters to build the networks, which can be used depending on the type and amount of data to be analyzed. Though, in general, a few steps may be considered: if working with (i) large datasets or organisms with huge interaction databases (Table S4), the network size can be reduced by using the Class score or FSW score filters; (ii) small datasets, the network can give more information when expanded to second or third neighbors; (iii) organisms for which only a few interactions were described, the network can be built by using the “ortholog relationship” option selecting a phylogenetically close model organism; (iv) transcriptome or proteome datasets, the network can be more coherent and concise by expanding it only to first neighbors and using the “delete nodes with degree 0 and 1” option; (v) metabolome datasets, an expansion to second neighbors may be more interesting, since it will probably allow clusters of metabolites and first neighbors to connect with each other; and (vi) drugs datasets, the same as for metabolome datasets.

Therefore, from the analyses presented above, IIS comes as a platform to perform an integrative analysis of omics data focused on interaction networks, mainly visualized via web or by Cytoscape software, in a more complete and easy-to-interpret way, in order to give a first overview of all the components, their emergent properties and relations and assist researchers to direct further relevant experiments and take important insights of their data. IIS is freely available online at: http://www.lge.ibi.unicamp.br/lnbio/IIS/. IIS code and database can be downloaded at: http://bioinfo03.ibi.unicamp.br/lnbio/IIS2/download.php.

## Supporting Information

Table S1 **Automatic annotation table generated from the Annotation Module.**
(XLS)

Table S2 **Cellular component (GO) used in our database.** Children terms were grouped for each selected ancestral cellular component, considering only terms annotated for ≥10 genes.
(XLS)

Table S3 **Node attributes from hNek6 second neighbors network.** All attributes are described in details in Methods S1. Enriched GO Biological Processes (BP) and KEGG Pathways are depicted with a p-value in parentheses for each protein in the network. Only enriched terms with p≤0.05 were considered in the network analyses. hNek6 interactors retrieved by yeast two-hybrid are depicted in bold. Nodes with degree 0 and 1 were deleted from the network.
(XLS)

Table S4 **Statistics from GPMGDID.**
(XLS)

Methods S1 **GPMGDID construction, IIS pipeline and XGMML file generation.**
(PDF)
