# Correction: IIS – Integrated Interactome System: A Web-Based Platform for the Annotation, Analysis and Visualization of Protein-Metabolite-Gene-Drug Interactions by Integrating a Variety of Data Sources and Tools

**DOI:** 10.1371/journal.pone.0108101

**Published:** 2014-09-19

**Authors:** The PLOS ONE Staff

In the Author Contributions section, Gabriela Vaz Meirelles (GVM) should be listed as one of the persons who performed the experiments.

There is an error in the title of Figure 1. The complete, correct Figure 1 title is: Workflow used in IIS, showing the integration of the (1) SUBMISSION, (2) SEARCH, (3) ANNOTATION and (4) INTERACTOME MODULES for data analysis.

There is an error in the third sentence of the first paragraph of the “Second case study: *S. cerevisiae* encapsulated cells proteome” subsection of the Results and Discussion. Table S1 incorrectly links to the article’s supporting information. The reference Table S1 can be found in the paper Westman JO, Taherzadeh MJ, Franzén CJ (2012) Proteomic analysis of the increased stress tolerance of saccharomyces cerevisiae encapsulated in liquid core alginate-chitosan capsules. PLoS One 7 (11): e49335. doi: 10.1371/journal.pone.0049335.

There is an error in the second sentence of the first paragraph of the “Third case study: primary and metastatic human ovarian cancer metabolome” subsection of the Results and Discussion. Table S2 incorrectly links to the article’s supporting information. The reference Table S2 can be found in the paper Fong MY, McDunn J, Kakar SS (2011) Identification of metabolites in the normal ovary and their transformation in primary and metastatic ovarian cancer. PLoS One 6 (5): e19963. doi: 10.1371/journal.pone.0019963.
