# Supplementary material for: IIS – Integrated Interactome System: A Web-Based Platform for the Annotation, Analysis and Visualization of Protein-Metabolite-Gene-Drug Interactions by Integrating a Variety of Data Sources and Tools
Source: PLoS One. 2014 Jun 20;9(6):e100385. doi: 10.1371/journal.pone.0100385 (PMC4065059; doi:10.1371/journal.pone.0100385)
Supplement: Methods S1 — GPMGDID construction, IIS pipeline and XGMML file generation. (PDF) [file pone.0100385.s005.pdf]

Methods S1

Supporting Information to:

IIS – Integrated Interactome System: a Web-Based Platform for the Annotation, Analysis and Visualization of Protein-Metabolite-Gene-Drug Interactions by Integrating a Variety of Data Sources and Tools

Marcelo Falsarella Carazzolle[1,2], Lucas Miguel de Carvalho[1], Hugo Henrique Slepicka[3], Ramon Oliveira Vidal[2], Gonçalo Amarante Guimarães Pereira[2], Jörg Kobarg[1], Gabriela Vaz Meirelles[1*]

[1] Laboratório Nacional de Biociências, Centro Nacional de Pesquisa em Energia e Materiais, Campinas, SP, Brazil

[2] Laboratório de Genômica e Expressão, Departamento de Genética e Evolução, Instituto de Biologia, Unicamp, Campinas, SP, Brazil

[3] Laboratório Nacional de Luz Síncrotron, Centro Nacional de Pesquisa em Energia e Materiais, Campinas, SP, Brazil

[*] corresponding author

GPMGDID construction

Organism classification

All the organisms containing interactions described in the public databases used to build the Global Protein-Metabolite-Gene-Drug Interaction Database (GPMGDID) were also considered in the GPMGDID integration. The difference is that in our integrated database the organisms were classified into species and all subspecies had their taxonomic IDs converted to their corresponding species IDs in order to group them into

their species names and IDs (Table S4). This allows building more complete networks by species since protein-protein interactions studied in a particular subspecies are added into the generated network of that species. Though, if the user wants to see only the interactions for that specific subspecies, the original taxonomic ID of each protein is also available in the generated XGMML file, and so the user can easily filter this information in the annotation table inside Cytoscape (Data Panel).

Integration of experimental interaction data

GPMGDID is a non-redundant database which integrates all protein-metabolite-gene-drug interactions described in several public databases and for several organisms. Interaction pairs are classified by experimental methodology (e.g. two-hybrid, pull down, genetic interference, etc.), organism and source literature (PubMed ID of the paper in which the interaction was described), while the proteins/genes involved in the interaction are characterized by Gene Ontology annotation database (biological process, molecular function and cellular component) and KEGG pathways allowing the compartmentalization and enrichment analysis performed in the INTERACTOME MODULE.

The experimental information about protein-metabolite-gene-drug interactions can be accessed on several public databases but there is no standardization of protein identifiers and experimental methods description hindering the integration steps. Although there are some efforts to construct standardized databases, like the PSI-MI TAB format for protein-protein interaction and JSON format for small molecules-proteins databases, many other problems related to different protein identifiers (UniProt ID, gene symbol, Ensembl ID or RefSeq) and source literature (PubMed ID or DOI) need to be solved in order to organize these information as an integrated database. GPMGDID database and scripts were developed to solve these problems as described below (Figure S1).

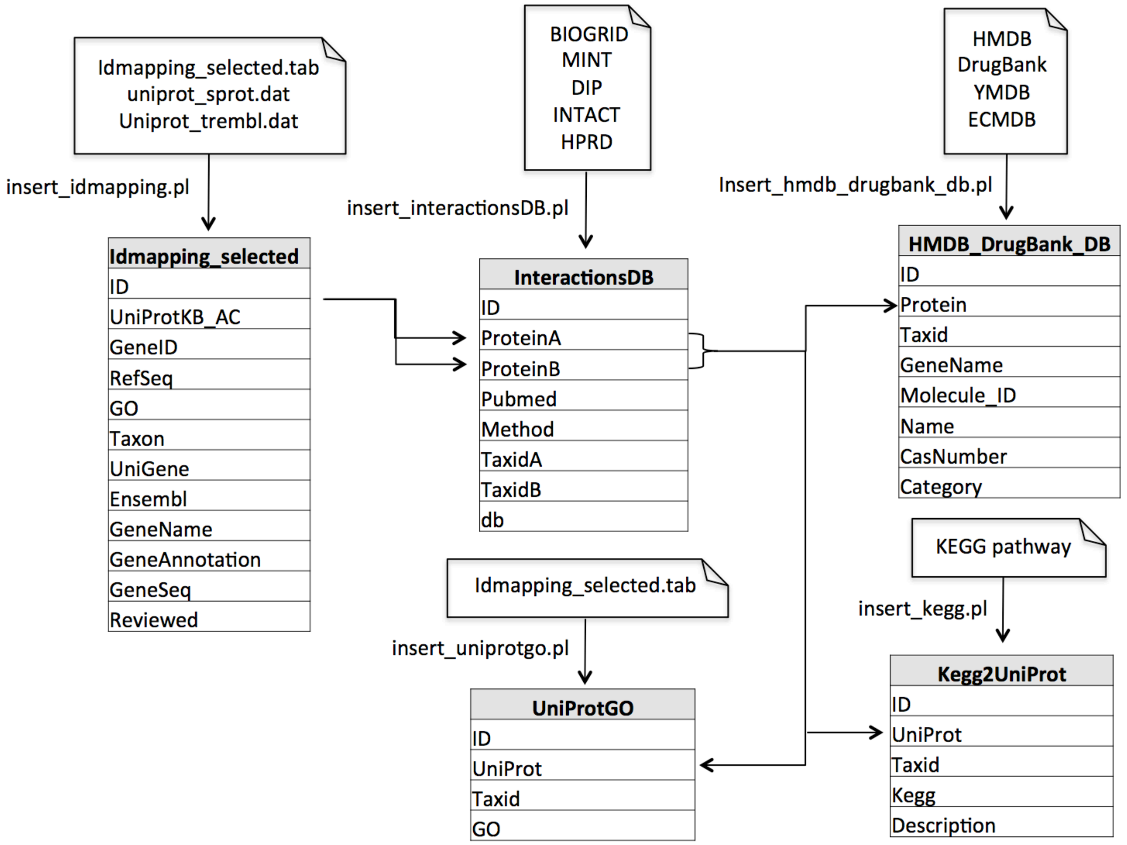

Figure S1. GPMGDID construction pipeline showing information about flat input files and PERL scripts used to obtain a non-redundant, standard and integrated database.

In order to do the correlation of several protein identifiers, a database named Idmapping_selected was constructed containing all protein identifiers used in these different public databases. The Database Identifier Mapping (idmapping_selected.tab; ftp://ftp.uniprot.org/pub/databases/uniprot/current_release/knowledgebase/idmapping/) and the UniProtKB/Swiss-Prot together with the TrEMBL annotation files (ftp://ftp.uniprot.org/pub/databases/uniprot/current_release/knowledgebase/complete/), both available at UniProt [44] website, were used as input to homemade PERL script (insert_idmapping.pl) that creates the database table considering UniProtKB_AC as the unique protein identifier. The fields named GeneSeq and Reviewed contain information about protein sequences and manually curated annotations that are used in the ANNOTATION and INTERACTOME MODULES of IIS.

The PERL scripts insert_interactionsDB.pl and insert_HMDB_drugbank_db.pl were developed to parse the information available in public interaction databases

(BioGRID [9], Intact [10], DIP [11], MINT [13], HPRD [14], DrugBank [15], HMDB [17], YMDB [18], ECMDB [19]) and construct the database tables InteractionsDB and HMDB_DrugBank_DB which contain information related to non-redundant protein-protein interactions and metabolite/drug-protein interactions, respectively. In order to eliminate redundant information produced by the same interaction pair described in more than one public database, the scripts insert the interaction pair into the table only if an interaction pair ID given by UniProt_ID1_UniProtID2_PubMedID does not exist. Also, these scripts convert the protein identifiers to UniProtKB_AC using the Ipmapping_selected table, and if there is more than one protein matching to the same UniProtKB_AC the reviewed entry (manually curated from Swiss-Prot database) is prioritized. As described in the "Organism classification" section above, the taxonomic IDs for subspecies were converted to their corresponding species ID (Table S4), and the insert_interactionsDB.pl and insert_HMDB_drugbank_db.pl scripts use this information to save the interaction pair into GPMGDID database to maintain the taxonomic ID in the species level.

Finally, the UniProtGO and Kegg2UniProt tables contain 1:N relationships between UniProtKB_AC and Gene Ontology IDs or UniProtKB_AC and KEGG pathways, respectively. These tables are created by the insert_uniprotgo.pl and insert_kegg.pl scripts and used to perform the analyses of protein/gene compartmentalization and enrichment in the INTERACTOME MODULE.

IIS pipeline

The Integrated Interactome System (IIS) integrates four different modules for processing, annotation, analysis and visualization of the interaction profiles. The system accepts three inputs of data types: chromatograms, protein/gene lists and metabolite/drug lists. Figure S2 shows the pipeline constructed to process these datasets in order to connect them to the GPMGDID database through the INTERACTOME MODULE.

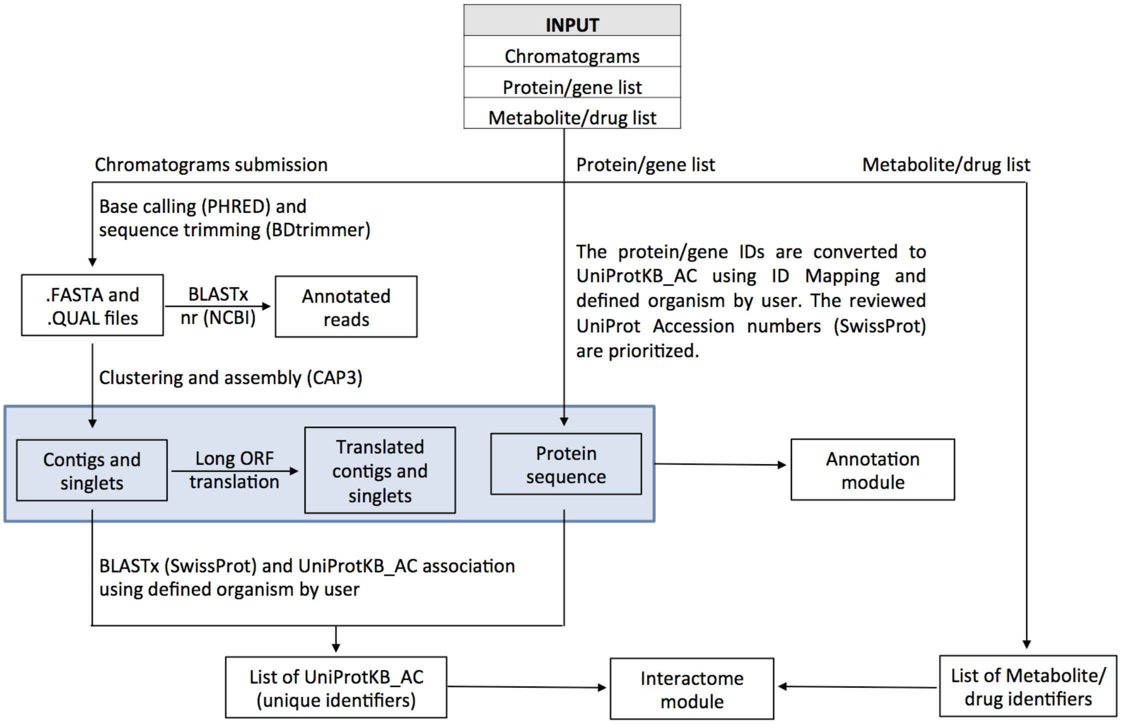

Figure S2. IIS pipeline showing the processing and integration of three input data types used in the IIS platform.

As shown in Figure S2, between the three types of input the chromatogram data are the most complicated to process since they require several processing steps: chromatogram submission, chromatogram processing, reads annotation, reads clustering/assembly and contigs annotation. All results obtained from each step can be accessed by the user through the IIS interface. The chromatogram submission receives the uploaded chromatograms file in a ZIP format (each file containing from one to 96 chromatograms named according to the position in the 96 well sequencing plates, e.g. A01 to H12), checks the file integrity of the ZIP file and the individual chromatograms after uncompressing, organizes the uncompressed chromatograms in a directory structure (chromat_dir, edit_dir and phd_dir) to enable the execution of PHRED and BDTrimmer programs, sends an email to the user summarizing the information about the chromatograms processing, and finally starts the reads annotation step blasting the trimmed reads sequences against GenBank/NR (NCBI) protein database. All these steps are performed in the SUBMISSION MODULE. The reads annotation step performs only a partial annotation (BLASTx against GenBank/NR (NCBI) with e-value threshold of 1e-

10) allowing the user to verify the protein identity and homologous organism of each read. In the case of chromatograms submission which *Homo sapiens* is the selected organism (organism = HS in the Nomenclature field of the SUBMISSION MODULE), the reads annotation pipeline prioritizes the best RefSeq alignment of an *Homo sapiens* hit. The reads partial annotation together with the submission report can be useful for the user to evaluate the quality of the yeast two-hybrid (Y2H) experiments and sequencing. Finally, in order to eliminate the redundant reads typically generated by Y2H and transcriptome assays, the reads are assembled into clusters (contigs and singlets) using CAP3 program with default parameters (overlap length cutoff ≥ 40 and overlap percent identity cutoff ≥ 80).

Annotation table creation

Nine databases were downloaded to be used in the ANNOTATION MODULE: Gene Ontology [35], HPA (Normal tissue data, Cancer tumor data and RNA data) [36], CDD [37], MGI [38], PDB [39], DisEMBL [40], Prosite [41], Ensembl [42] and Swiss-Prot [43]. The information related to UniProt Accession numbers, Gene Ontology and Ensembl of proteins/genes and contigs/singlets are obtained through searching against our ID mapping using UniProtKB_AC as a unique identifier. For the contigs/singlets a previous step is necessary in which the contigs/singlets sequences are blasted (BLASTx) against the Swiss-Prot database [43] with an e-value threshold of 1e-20, and the association between contigs/singlets and UniProtKB_AC is performed by using the best alignment hit containing a sequence identity ≥ 30%. Here users can both choose to blast their sequences against the complete Swiss-Prot database, by selecting the “All” option in the Organism field in the Annotation Builder window, or blast against the Swiss-Prot database restricted to one specific organism. The annotation process for CDD (ftp://ftp.ncbi.nlm.nih.gov/pub/mmdb/cdd) and PDB (ftp://ftp.ncbi.nlm.nih.gov/blast/db/pdbaa) is based on sequence similarity using BLAST against these protein databases with e-value thresholds of 1e-10 and 1e-20, respectively. In the case of CDD, the RPSBLAST program was used for querying. The information related to Prosite [41] and DisEMBL [40] databases was based on protein sequence using ScanProsite (http://prosite.expasy.org/scanprosite) and disembl.py programs executed as

default parameters. Again, for the contigs/singlets, it is necessary to perform a previous step where the nucleic acid sequences are translated to amino acids sequences using longorf methodology (the nucleotide sequence is examined in the six frames for the longest ORF; the canonical ATG or STOP is not required enabling to start at the beginning and the end of the sequence). Finally, the HPA [36] and MGI [38] annotation were obtained by searching in tab-delimited flat files of Human Protein Atlas (normal_tissue.csv, cancer.csv and rna.csv; downloaded from http://www.proteinatlas.org/about/download) and MGI database (MRK_Ensembl_Pheno.rpt; downloaded from ftp://ftp.informatics.jax.org/pub/reports/index.html).

The retrieved information from each database used to create the annotation tables are as follows: gene symbol, protein description and organism (Swiss-Prot); PDB ID and protein description (PDB); CDD ID and conserved domain name and description (CDD); Prosite ID and motif term (Prosite); GO ID and cellular component, molecular function and biological process terms (Gene Ontology); Coils, Remark465 and Hot loops percentage of disordered residues (DisEMBL); MP ID and mammalian phenotype terms (MGI); tissue, cell type, level, expression type and condition terms (HPA). For GO, DisEMBL, MGI and HPA only the top 30 results from each database are shown in the table. The percentage of disordered residues was calculated dividing the number of disordered residues assigned by Coils, Remark465 and Hot loops predictors of DisEMBL by the total number of residues of the protein.

Integration with the GPMGDID database

The fundamental point that enables the integration between IIS pipeline and GPMGDID database is the use of UniProtKB_AC as the unique identifier, so both the contigs/singlets and protein/gene lists must be converted to UniProtKB_AC, as explained above. For the contigs/singlets, their sequences are blasted (BLASTx) against the Swiss-Prot database [43] with an e-value threshold of 1e-20, and the association between contigs/singlets and UniProtKB_AC is performed by using the best alignment hit containing a stringent sequence identity ≥ 95%. In the case of protein/gene input data the user can either submit a list of Gene Symbol, UniProtKB_AC or Refseq as the

protein/gene identifier, so the conversion from Gene Symbol and Refseq to UniProtKB_AC is necessary and can be obtained through searching against our ID mapping database (Ipmapping_selected table) considering the organism defined by the user and choosing a reviewed entry if there is more than one protein matching to the same ID in the database.

For the metabolite/drug list, there is a complete equivalence between the metabolite/drug IDs accepted as input for IIS pipeline and used in the GPMGDID database.

Interactome (XGMML file) generation

Experimental methods for the detection of types of interactions

The classification of interactions in protein-protein (pp), protein-gene (pg) and gene-gene (gg) interactions in the network is based on the method by which the interactions have been detected. The list of experimental methods from each database that comprise GPMGDID were analyzed, and the following methods were considered to identify pg and gg types of interactions: "chromatin immunoprecipitation array", "chromatin immunoprecipitation assay", "chromatin immunoprecipitation assays", "DNase I footprinting", and "one hybrid" to characterize pg, and "genetic interference" to characterize gg. The remaining methods were considered to characterize pp. Interactions between proteins and metabolites or proteins and drugs were classified as protein-metabolite (pm) and protein-drug (pd) interactions, respectively.

Nodes and edges attributes

Nodes and edges are annotated in the generated network according to diverse attributes that can be used to cluster nodes or compare different networks. These attributes are defined as follows:

Node attributes:

- ID: gene, metabolite or drug name registered in our database.
- UniProt ID: gene/protein entry defined in the UniProt database.
- TaxID: taxonomic identifier.

- Gene: gene name registered in our database according to UniProt.
- Metabolite: metabolite name registered in our database according to HMDB, YMDB or ECMDB.
- Metabolite ID: metabolite identifier registered in our database according to HMDB, YMDB and/or ECMDB.
- Drug: metabolite name registered in our database according to DrugBank.
- Drugbank ID: metabolite identifier registered in our database according to DrugBank.
- p-value: p-value calculated for each node to be included in the network (the default is to include nodes with p-value ≤ 0.05).
- node.label: gene, metabolite or drug name registered in our database, which can be modified when using Cytoscape.
- node.shape: shape of each type of node. We use circle ("ellipse" in the xgmml) for genes/proteins, square ("rectangle" in the xgmml) for metabolites, and triangle ("triangle" in the xgmml) for drugs.
- node.fillColor: color of each node defined by values in the RGB scale. The default are: input contigs/singlets or genes/proteins from the project = blue; metabolites/drugs = yellow; bait (if applicable) = red; first neighbors = green; second neighbors = orange; third neighbors = purple. If selecting node color to be relative to fold change (FC) values, the molecules node colors will be defined by the following relations: up-regulated molecules = red; down-regulated molecules = green; non-regulated molecules = yellow; first/second/third neighbors = gray.
- node.size: size of each node. The default gene/protein node sizes are defined by the equation: node.size = degree + 20. The default metabolite and drug node sizes are equal to 20. If selecting node size to be relative to fold change (FC) values, the molecules node sizes will be defined by linear relationships, considering three FC ranges: 1 – 10, 10 – 100 and 100 – 1000.
- Degree (pp): degree connectivity of each node (the number of neighbors of a node), considering only interactions between genes/proteins (pp = protein-protein).

- Biological Process (GO): all the terms separated by “;” that define the biological processes of each gene/protein according to Gene Ontology (GO).
- Cellular Component (GO): all the terms separated by “;” that define the cellular component of each gene/protein according to Gene Ontology (GO). Several children terms were grouped in the same ancestral term in order to have a more concise list of the main cellular compartments (Table S2).
- Selected CC: a unique term selected from the “Cellular Component (GO)” attribute for each gene/protein to be used to separate the nodes in an easier-to-visualize and interpret layout. This term is selected according to the following order of priority: extracellular > cell wall > plasma membrane > mitochondrion > endoplasmic reticulum > golgi apparatus > endosome > centrosome > microtubule organising centre > lysosome > vacuole > glyosysome > glycosome > peroxisome > amyloplast > apicoplast > chloroplast > plastid > cytosol > cytoplasm > nucleus (Table S2). Endosome, centrosome, microtubule organising centre, lysosome, vacuole, glyosysome, glycosome, peroxisome, amyloplast, apicoplast, chloroplast, plastid, cytosol and cytoplasm > nucleus, only if **not** annotated with the following GO molecular function terms: GO:0000496 (base pairing), GO:0003677 (DNA binding), GO:0000497 (base pairing with DNA), GO:0003684 (damaged DNA binding), GO:0008301 (DNA binding, bending), GO:0003689 (DNA clamp loader activity), GO:0010844 (recombination hotspot binding), GO:0000975 (regulatory region DNA binding), GO:0043565 (sequence-specific DNA binding), GO:0043566 (structure-specific DNA binding), GO:0044212 (transcription regulatory region DNA binding), GO:0031490 (chromatin DNA binding), GO:0045027 (DNA end binding), GO:0097100 (supercoiled DNA binding), GO:0003697 (single-stranded DNA binding, GO:0003690 double-stranded DNA binding), GO:0000988 (protein binding transcription factor activity), GO:0000990 (core RNA polymerase binding transcription factor activity), GO:0000989 (transcription factor binding transcription factor activity), GO:0000996 (core DNA-dependent RNA polymerase binding promoter specificity activity), GO:0001181 (core RNA polymerase I binding transcription factor activity), GO:0000991 (core RNA

polymerase II binding transcription factor activity), GO:0000995 (core RNA polymerase III binding transcription factor activity), GO:0043856 (anti-sigma factor antagonist activity), GO:0001082 (RNA polymerase I transcription factor binding transcription factor activity), GO:0001076 (RNA polymerase II transcription factor binding transcription factor activity), GO:0001007 (RNA polymerase III transcription factor binding transcription factor activity), GO:0016989 (sigma factor antagonist activity), GO:0003712 (transcription cofactor activity), GO:0001134 (transcription factor recruiting transcription factor activity), GO:0001104 (RNA polymerase II transcription cofactor activity), GO:0003713 (transcription coactivator activity), GO:0003714 (transcription corepressor activity), GO:0001186 (RNA polymerase I transcription factor recruiting transcription factor activity), GO:0001135 (RNA polymerase II transcription factor recruiting transcription factor activity), GO:0001153 (RNA polymerase III transcription factor recruiting transcription factor activity), GO:0001010 (sequence-specific DNA binding transcription factor recruiting transcription factor activity), GO:0001026 (TFIIIB-type transcription factor activity), GO:0001083 (RNA polymerase II basal transcription factor binding transcription factor activity), GO:0001191 (RNA polymerase II transcription factor binding transcription factor activity involved in negative regulation of transcription), GO:0001190 (RNA polymerase II transcription factor binding transcription factor activity involved in positive regulation of transcription), GO:0001071 (nucleic acid binding transcription factor activity), GO:0003700 (sequence-specific DNA binding transcription factor activity), GO:0001099 (basal RNA polymerase II transcription machinery binding), GO:0001091 (RNA polymerase II basal transcription factor binding), GO:0000993 (RNA polymerase II core binding), GO:0001092 (TFIIA-class transcription factor binding), GO:0001093 (TFIIB-class transcription factor binding), GO:0001094 (TFIID-class transcription factor binding), GO:0001095 (TFIIE-class transcription factor binding), GO:0001096 (TFIIF-class transcription factor binding), GO:0001097 (TFIIH-class transcription factor binding), GO:0001042 (RNA polymerase I core binding), GO:0000994 (RNA polymerase III core binding).

- Enriched BP: all the terms separated by ";" that define the biological processes of each gene/protein according to Gene Ontology (GO), which are enriched in the network (p-value ≤ 0.05), and the respective p-values in parentheses.
- Enriched KEGG: all the terms separated by ";" that define the metabolic and signaling pathways of each gene/protein according to KEGG, which are enriched in the network (p-value ≤ 0.05), and the respective p-values in parentheses.
- Top Enriched BP: the most enriched biological process (with the lowest p-value) for each gene/protein.
- p-value (Top Enriched BP): the corresponding p-value of the "Top Enriched BP" attribute.
- Top Enriched KEGG: the most enriched pathway (with the lowest p-value) for each gene/protein.
- p-value (Top Enriched BP): the corresponding p-value of the "Top Enriched KEGG" attribute.

Edge attributes:

- ID: interaction name defined by the gene, metabolite or drug names registered in our database that interact to each other (e.g. ProteinA (pp) ProteinB).
- interaction: type of the interaction (pp = protein-protein, pm = protein-metabolite, pd = protein-drug, pg = protein-gene, gg = gene-gene).
- PubMed ID: identifier of the paper that describes the interaction.
- Method: method used to identify the interaction.
- Cellular Component (GO): intersection between the cellular components described for each node of the interacting pair of nodes.
- FSW score: Functional Similarity Weight score (from 0 to 10) which measures how similar two interacting nodes are, based on how many neighbors a pair of nodes share.
- Class score: score (from class A to E) which measures how confident an interaction is, based on the number of papers that describe the interaction, if the interacting nodes are described in the same cellular compartment and if it has experimental evidence.
